# Bipolar versus fixed-head hip arthroplasty for femoral neck fractures in elderly patients

**DOI:** 10.1007/s11751-010-0100-1

**Published:** 2011-01-22

**Authors:** Mostafa Abdelkhalek, Mohamed Abdelwahab, Ayman M. Ali

**Affiliations:** Department of Orthopedic Surgery, Mansoura University, Dakahliya, Egypt

**Keywords:** Femoral neck fractures, Hip prosthesis, Arthroplasty

## Abstract

Between 2002 and 2007, fifty elderly patients with displaced femoral neck fractures were treated with hip replacement at Emergency Hospital, Mansoura University. Patients were randomly selected, 25 patients had either cemented or cementless bipolar prosthesis, and another 25 patients had either cemented or cementless fixed-head prosthesis. There were 34 women and 16 men with an average age of 63.5 years (range between 55 and 72 years). All patients were followed up both clinically and radiologically for an average 4.4 years (range between 2 and 6 years). At the final follow-up, the average Harris hip score among the bipolar group was 92 points (range between 72 and 97 points), while the fixed-head group was 84 points (range between 65 and 95 points). Radiologically, joint space narrowing more than 2 mm was found in only 8% (2 patients) among the bipolar group, and in 28% (7 patients) of the fixed-head group. Through the follow-up period, total hip replacement was needed in two cases of the bipolar group and seven cases of the fixed-head group. Bipolar hemiarthroplasty offered a better range of movement with less pain and more stability than the fixed-head hemiarthroplasty in elderly patients with displaced femoral neck fractures.

## Introduction

Since displaced intracapsular femoral neck fractures have a significant risk of nonunion and avascular necrosis, prosthetic replacement is often recommended in ambulatory, elderly patients [1]. The fixed-head hemiarthroplasty is associated with high acetablular erosion and protrusion rates, which affect the clinical results and makes revision to a total hip arthroplasty difficult [2].

These complications have led many surgeons to choose a bipolar design. The theoretical advantage of a bipolar hemiarthroplasty is to decrease acetabular erosion and wear and their associated symptoms [3]; however, there is still some debate concerning the benefits of bipolar versus the fixed-head hemiarthroplasty [4]. The aims of this study are to evaluate the results of bipolar versus fixed-head hemiarthroplasty for displaced femoral neck fractures in elderly patients and to address the problems of prosthesis selection.

## Patients and methods

Fifty elderly patients with displaced femoral neck fractures were treated at Emergency Hospital, Mansoura University with hemiarthroplasty. There were 34 women and 16 men. The average age at operation was 63.5 years (range 55–72 years). Patients were allocated randomly, with alternate cases undergoing a bipolar or fixed-head hemiarthroplasty. Surgery was performed in all cases through the posterior approach of the hip. The method of prosthesis fixation (either cemented or cementless) was selected intra-operatively depending on the quality of bone and the presence or absence of a sufficient calcar of the femoral neck. Cement was introduced using a cement gun. In the bipolar group, 12 patients had a cemented prosthesis while 13 patients had a cementless prosthesis. In the fixed-head group, 15 patients had a cemented Thompson prosthesis and 10 patients had a Austin Moore’s prosthesis (Table 1).

**Table 1:** Types of prosthesis

Types	Number	%
Cemented bipolar	12	48
Cementless bipolar	13	52
Austin Moore’s prosthesis	10	40
Cemented Thompson	15	60
Total	50	100

All patients had prophylactic low molecular weight heparin 12 h pre-operatively and daily post-operatively for 5 days. Ambulation with weight bearing as tolerated was started on the second or third post-operative day.

All patients were followed up and evaluated clinically and radiologically post-operatively at 6 weeks, 3 months, 6 months, and at 1 year and then annually.

Clinically, hip function was evaluated using the Harris hip score with a total score of 100 points according to the presence or absence of pain, the use of support, the distance walked, the presence or absence of limp, activities, the manner of using stairs, public transportation, sitting, deformities, and the range of motion. In the Harris hip score, a total score above 90 points is an excellent result, 80–90 points is a good result, 70–80 points is a fair result, and below 70 points is a poor result.

**Fig. 1:**
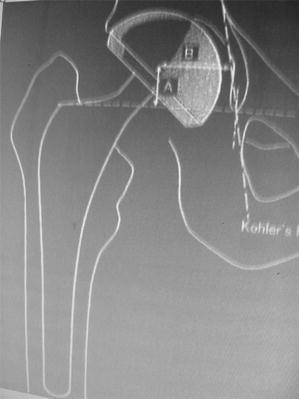
Measurement of outer head migration (Murzic and McCollum) [5].*A* Superior migration is assessed by measuring the distance between the center of the outer head and the inferior margin of the ipsilateral tear drop. *B* Medial migration is determined by measuring the distance from Kohler’s line and the center of the outer head

Radiological evaluation included antero-posterior and lateral views. Migration of the prosthesis in the acetabulum was defined by the method of Murzic and McCollum [5], which included medial migration and superior migration. Superior migration is assessed by measuring the distance between the center of the outer head and the inferior margin of the ipsilateral tear drop. Medial migration is determined by measuring the distance from Kohler’s line and the center of the outer head (Fig. 1).

Acetabular cartilage erosion was determined by measuring the change in thickness of the acetabular cartilage compared with the immediate post-operative film. Femoral component subsidence was determined by comparing measurements from the prosthesis collar to the lesser trochanter as described by Gingras et al. [6]. Radiographic femoral loosening was recorded by measuring radiolucencies at the prosthesis–cement or cement–bone interface in the seven zones described by Gruen et al. [7].

## Results

The duration of follow-up ranged between 2 and 6 years with an average of 4.4 years. The overall results showed a statistical significant favorable results for the bipolar group over the fixed-head group (*P* = 0.004). The average Harris hip score for the bipolar group was 92.3 points (range 72–97 points) with 44% of cases had excellent results, 2% had good results, and 4% had fair results, while in the fixed hip group, the average Harris hip score was 84.3 points (range 65–95 points) with only 20% of cases had excellent results, 16% had good results, 10% had fair results, and 4% had poor results (Table 2).

**Table 2:** Type of prosthetic replacement and Harris hip score

		Harris hip score				χ^2^ test	*P* value
		Excellent	Good	Fair	Poor		
Types of prosthetic replacement	Bipolar hemiarthroplasty (*n* = 25)	22 (44.0%)	1 (2.0%)	2 (4.0%)	0 (0.0%)	13.23	0.004
	Fixed-head hemiarthroplasty (*n* = 25)	10 (20.0%)	8 (16.0%)	5 (10.0%)	2 (4.0%)		

Acetabular cartilage erosion and joint space narrowing was found in 14% of cases of fixed hip group and 4% of the bipolar group with a statistically significant difference (*P* < 0.05) (Table 3).

**Table 3:** Type of prosthetic replacement and joint space narrowing

			Joint space narrowing >2 mm		Total
			Yes	No	
Types of prosthetic replacement	Bipolar hemiarthroplasty	Count	2	23	25
		% of total	4.0	46.0	50.0
	Fixed-head hemiarthroplasty	Count	7	18	25
		% of total	14.0	36.0	50.0
Total		Count	9	41	50
		% of total	18.0	82.0	100.0

In the bipolar group, the superior and medial migrations in the acetabulum were 0–1.6 mm (average 0.5 mm) and 0–1.0 mm (average 0.7 mm), respectively. In the fixed-head group, they were 0–12 mm (average 3.4 mm) and 0–8 mm (average 3.0 mm), respectively. There was a statistical significance between both groups in the superior and medial migration (*P* < 0.05).

**Fig. 2:**
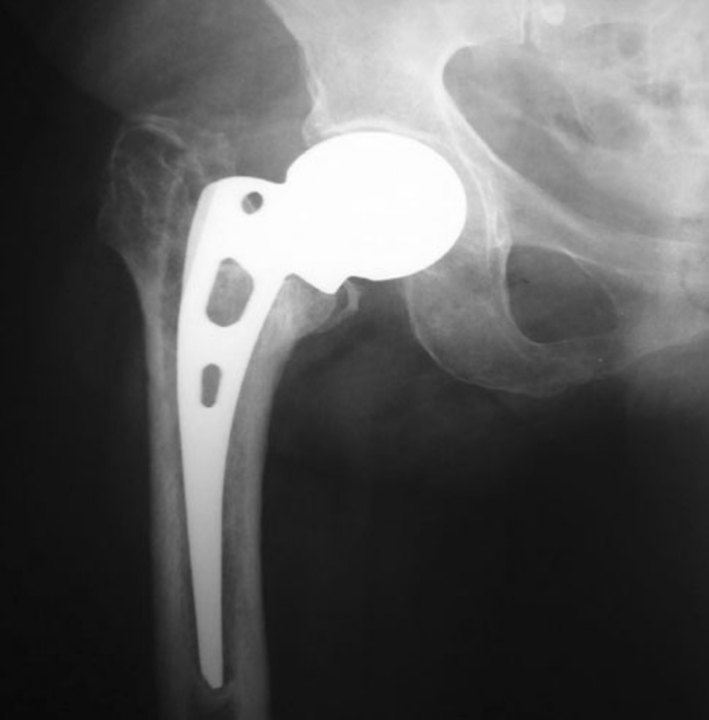
A 64-year-old patient with fracture neck femur treated with Austin Moore arthroplasty. Two years post-operatively radiograph showed subsidence of the prosthesis

**Fig. 3:**
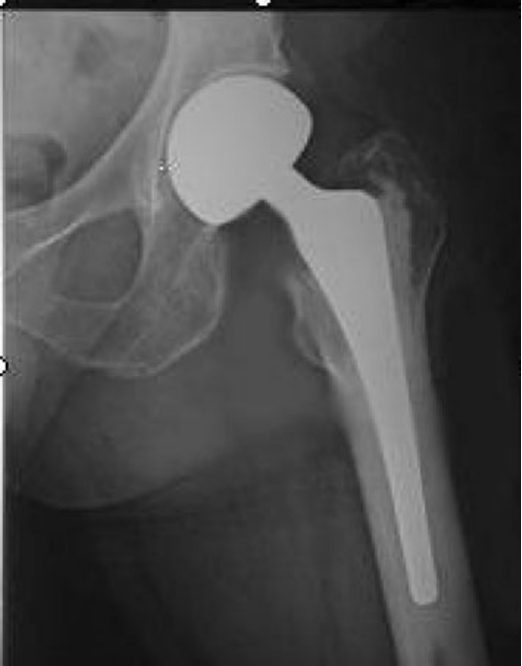
A 67-year-old patient with fracture neck femur treated with bipolar hip arthroplasty. At the final follow-up radiograph showing the outer head of the prosthesis in the anatomic position, articular cartilage space has been preserved, and neither migration of the outer head nor loosening of the femoral stem was seen

Calcar resorption (subsidence) was noted as early as 4 months post-operatively in three cases of Austin Moore prosthesis (Fig. 2). Two cases of acetabular protrusion occurred in the fixed-head group, while no case of protrusion in the bipolar group (Fig. 3).

There was a better range of movement in the bipolar group than the fixed-head group (Table 4). Limb length discrepancy less than 3.2 cm (1.5 inches) was found in one case (2%) of bipolar group and six cases (12%) with the fixed-head group. Through the follow-up period, total hip replacement was needed in two cases of the bipolar group and seven cases of the fixed-head group. The most common cause of failure leading to revision was aseptic femoral loosening.

**Table 4:** Range of motion

	Bipolar [25]		Fixed-head [25]	
	Mean	SD	Mean	SD
Flexion	94.68	2.39	83.04	2.11
Abduction	15.52	0.65	10.36	0.75
Adduction	14.04	0.67	10.32	0.94
External rotation	13.96	0.73	9.72	0.84

As regards, hip pain, in the bipolar group hip pain was absent in 17 (68%) patients, 6 (24%) had slight pain occasionally, and 2 (8%) patients had mild-to-moderate hip discomfort. In the fixed-head group, 7 patient (28%) had no pain, 12 (48%) had slight occasional pain, 5 patients (20%) had moderate pain, and only 1 patient (4%) had severe disabling pain (Table 5). When comparing hip pain in bipolar group versus fixed-head group, the bipolar group had less pain than fixed-head group with a statistically significant value (*P* = 0.038). Cemented group also had less pain than noncemented group with a statistically insignificant value (*P* = 0.453) (Table 6).

**Table 5:** Hip pain in bipolar and fixed-head groups

Pain score	Bipolar [25]		Fixed-head [25]		χ^2^	*P* value
	*N*	%	*N*	%		
Non	17	68.0	7	28.0	8.452	0.038
Slight	6	24.0	12	48.0		
Mild to moderate	2	8.0	5	20.0		
Severe	0	0.0	1	0.0

**Table 6:** Hip pain in cemented and noncemented groups

Pain score	Cemented [22]		Noncemented [28]		χ^2^	*P* value
	*N*	%	*N*	%		
Non	13	59.1	11	39.3	2.627	0.453
Slight	6	27.3	12	42.9		
Mild	3	13.6	4	14.3		
Severe	0	0.0	1	3.6		

In the bipolar group, 90% of the patients had no limping and 10% had a slight limping, while in the fixed-head group 22% of the patients had a moderate-to-sever limping.

There was only one early dislocation that occurred 2 weeks after surgery in a case of cemented Thompson prosthesis, which was treated by open reduction and revision to a total hip arthroplasty was done. The cause of dislocation in this case was excessive retroversion of the prosthesis. Heterotopic ossification was present in one case of bipolar group. Superficial wound infection was encountered in two cases (4%), and both were treated with culture-based antibiotics and frequent dressing. There were no cases of deep infection or D.V.T.

Regarding activity of the patients, 90% of the patients with bipolar prosthesis returned to their pre-injury level of activity, in comparison with 70% in the fixed-head group with a statistically significance value (*P* = 0.04).

## Discussion

A bipolar hemiarthroplasty design has been used for the treatment of femoral neck fractures for more than 30 years. The proposed advantages of using the bipolar design rather than the conventional fixed-head designs for femoral neck fracture in elderly patients are still controversial [8, 9].

In a study of forty cases of Austin Moore replacement done for femoral neck fractures over an average follow-up period of 26 months, Jadhav et al. [10] reported a high incidence of early postoperative pain of noninfective origin, which correlates well with osteolysis. Shortening was seen in 75% cases ranging from 1 to 7 cm. A limp was seen in 35 cases (87.5%) due to pain, shortening, or abductor muscle weakness. Radiological evidence of complications like sinking, protrusion, and calcar resorption, etc. was seen in majority of the cases. In Andersson’s series [11], only 6% cases walked without a limp. Sarmiento [12] in his post-mortems of 24 cases stated that there was “noticeable or excessive motion of stem in the canal and failure of cancellous bone to fill the entire fenestrations in the stem”. Also, in his study of 160 Moore and Thompson prostheses, Whittaker et al. [13] reported that 5% of the acetabula had protrusion and 25% had narrowing after one to 4 years; 24% had protrusion and 64% had narrowing after more than 5 years. Gingras et al. [6] studied cemented Thompson endoprostheses for femoral neck fracture over an average follow-up period of 17 months. Ninety-two percent had no or slight pain, but 8% had evidence of protrusion. Wetherell and Hinves [14] reported a rate of erosion of 11% with the cemented Thompson prosthesis.

Efthekar [15] stated “pressure brought by the femoral prosthesis upon the acetabular cartilage makes subsequent migration of the prosthesis inevitable.”

The bipolar prosthesis has two bearing surfaces; load and frictional torque can theoretically be absorbed in part by the metal on polyethylene inner bearing reducing the magnitude of forces between the implant and acetabulam thus decreasing acetabular erosion [16]. Drinker and Murray [17] in a retrospective series compared the bipolar prosthesis with the Thompson prosthesis and could not show a significant advantage to the bipolar prosthesis. Calder et al. [18] in his study concluded that there is no justification for the use of the expensive bipolar hip prosthesis in femoral neck fracture. On the other hand, La Belle et al. [19] reported that bipolar prosthesis resulted in less pain and decreased protrusio in comparison with the conventional fixed-head prosthesis. Lestrange [20] reviewed 496 patients with bipolar replacements for displaced femoral neck fractures and compared them with patients having fixed-head prosthesis. He found that the bipolar prosthesis offered advantages over one piece designs in terms of stability, decreased acetabular erosion, and improved function.

In the current study, at the final follow-up, the overall results showed better results that were statistically significant for the bipolar group over the fixed-head group in Harris hip scores, acetabular erosion and protrusion. Our results disagree with the randomized prospective study of Van Thiel et al. [21] and Calder et al. [18], who did not find any differences between the Moore unipolar and bipolar prostheses concerning acetabular erosion. Our results are consistent with Yamagata et al. [22] and D’Arcy and Devas [23] who found more erosion with unipolar prosthesis, and Wetherell and Hinves [14] who reported a rate of erosion of 5.6% with the bipolar implant compared with 11% for the cemented Thompson prosthesis. Whittaker et al. [13] reported 5% of the acetabula protrusion after 1–4 year follow-up with Moore and Thompson prostheses. It was demonstrated in our series a lower incidence of acetabular protrusion in bipolar group in comparison with the fixed-head prosthesis. Micheal et al. [16] reported that the bipolar prosthesis reduces the acetabular shear forces through the use of an outer free acetabular cup that also articulates with a prosthetic femoral head. Sikorski [24] reported dislocation rates of 10% in the Thompson prostheses. LaBelle [19] in his study of bipolar hip arthroplasty for femoral neck fractures reported incidence of 0.8% dislocation. In our study, there was no dislocation of prosthesis in the bipolar group, while the incidence of dislocation with fixed-head prosthesis was 4%. This is consistent with Attarian et al. [25] who reported that bipolar prosthesis has a self-aligning acetabular component, which finds a correct orientation on its own (a self-centering mechanism), and the incidence of subluxation and dislocation is low.

The theoretical advantages of inner bearing motion of bipolar prosthesis have not been supported in the majority of the motion studies [1]. Verberne [26] reported that intraprosthetic motion was absent 3 months after surgery. Tsukamoto et al. [27] suggested that motion during walking occurred mainly at the outer bearing. Brueton et al. [28] showed that the size of the inner head was an important determination in allowing inner bearing motion. Small heads (22 mm) allowed bipolar motion, whereas large heads (32 mm) hindered inner bearing motion. The prostheses studied by Verberne [26] had a 32-mm head. Calder [18] reported that movement within the prosthesis may also reduce the pain caused by the prosthesis moving against the acetabulum. In our study, the bipolar group had a better range of motion and 90% of the patients had no limping and 10% had a slight limping, while in the fixed-head group, 22% of the patients had a moderate to severe limping. Favorable results of bipolar may be contributed by the fact that the modularity of the bipolar prosthesis allows for greater flexibility in “customizing” prosthetic sizing so that soft tissue tension and limb length equalization can be improved by ability to use variable neck lengths intra-operatively. This coincides with Cornell et al. [8] who reported that patients with bipolar prosthesis did better on walk tests and had better range of motion at 6 months.

From our results, the bipolar hemiarthroplasty seems to offer a better range of movement with less pain and more stability than the fixed-head hemiarthroplasty in elderly patients with displaced femoral neck fractures in spite of the increased cost factor.
